# Public Health Relevance of US EPA Air Quality Index Activity Recommendations

**DOI:** 10.1001/jamanetworkopen.2024.5292

**Published:** 2024-04-08

**Authors:** Robert D. Brook, Sanjay Rajagopalan, Sadeer Al-Kindi

**Affiliations:** 1 Division of Cardiovascular Diseases, Department of Internal Medicine, Wayne State University, Detroit, Michigan; 2 University Hospitals, Case Western Reserve University, Cleveland, Ohio; 3 DeBakey Heart and Vascular Center, Houston Methodist, Houston, Texas

## Abstract

**Importance:**

Reducing exposure to fine particulate matter (<2.5 μm [PM_2.5_]) air pollution improves cardiopulmonary morbidity and mortality. However, the public health relevance of air quality index (AQI) activity guidelines under present-day environmental conditions in the US has not been critically assessed.

**Objective:**

To evaluate the public health relevance of following PM_2.5_ AQI activity guidance in preventing serious atherosclerotic cardiovascular disease (ASCVD) and pulmonary events among adults in the US.

**Design, Setting, and Participants:**

This cross-sectional modeling study involved the general adult population and sensitive individuals as designated by the US Environmental Protection Agency (EPA), including adults with preexisting ASCVD or lung disease (asthma or chronic obstructive pulmonary disease). The study was conducted between August 1, 2023, and January 31, 2024.

**Exposures:**

Daily AQI strata for PM_2.5_ and the corresponding activity recommendations.

**Main Outcomes and Measures:**

The main outcome was the number needed to treat (NNT) per day by following activity guidance across daily AQI strata to prevent 1 serious ASCVD or pulmonary event among relevant populations. To calculate PM_2.5_-induced excess disease event rates per day, estimated baseline disease-specific daily event rates for each group were multiplied by the increase in risks due to PM_2.5_ levels at each AQI stratum. The number of events prevented per day was calculated by multiplying each excess disease event rate by the percentage in exposure reduction plausibly incurred by following population-specific activity guidance at each AQI level. The NNT is the reciprocal of the number of events prevented.

**Results:**

The NNT to prevent ASCVD events was high for the general population and for patients with ASCVD across all AQI strata. The range of values was comparatively lower to prevent pulmonary events among adults with lung disease. During most days (96%) when activity recommendations were promulgated due to elevated PM_2.5_ (AQI, 101-200), the NNT to prevent a serious disease event remained very high for the general population (>18 million), patients with ASCVD (approximately 1.6-5 million), and adults with lung disease (approximately 66 000-202 000).

**Conclusions and Relevance:**

These findings suggest that existing PM_2.5_ AQI activity recommendations are of questionable public health relevance in present-day conditions and merit consideration for updating to improve their potential effectiveness.

## Key Points

**Question:**

What is the public health relevance of US Environmental Protection Agency air quality index (AQI) activity guidance for exposure to fine particulate matter (<2.5 μm [PM_2.5_]) air pollution under present-day environmental conditions?

**Findings:**

This cross-sectional study modeled the number needed to treat (NNT) per day by following AQI activity guidance to prevent 1 serious atherosclerotic cardiovascular disease (ASCVD) or pulmonary event. The NNT remained high during most days (96%) when PM_2.5_ was elevated (AQI, 101-200) for the general adult population (>18 million), adults with ASCVD (approximately 1.6-5.0 million), and adults with pulmonary disease (approximately 66 000-202 000).

**Meaning:**

These findings suggest that updated strategies are needed to improve the public health relevance of AQI activity guidance in the US.

## Teaser

This cross-sectional study assesses the public health relevance of air quality index (AQI) activity guidance for exposure to fine particulate matter air pollution in preventing serious atherosclerotic cardiovascular disease and pulmonary events among US adults.

## Introduction

The US Environmental Protection Agency (EPA) has issued an air quality index (AQI) since 1976 to inform the public regarding ambient air pollution levels.^1^ The current version promulgated since 1999 additionally provides activity guidance related to lifestyle changes (eg, “avoid long or intense outdoor activities”) that may help reduce exposures. Specific recommendations are based on the AQI level and tailored to the risk of adverse health effects among various populations (eg, the general public and patients with cardiovascular disease).^2^ The main goal is to reduce air pollution–induced morbidity and mortality, most notably from cardiopulmonary diseases, which account for the largest portion of events.^3,4^ This general premise is plausible given the sound evidence linking reduced air pollution exposures with improved all-cause and cardiopulmonary mortality.^5,6,7,8^ To this end, daily AQIs (which are universally driven by fine particulate matter <2.5 μm [PM_2.5_] or ozone levels in the US) have undergone progressive updates to meet the increasingly stringent US National Ambient Air Quality Standards based on advancing scientific evidence over time.^1^ However, we are unaware of any critical assessment of the public health relevance of AQI activity recommendations in the past or, more importantly, under present-day environmental conditions, which have improved substantially over the prior few decades.^5,6,7,8,9,10^ As such, we aimed to help elucidate this issue by estimating the number of individuals needed to follow the activity recommendations for PM_2.5_ per day to prevent cardiopulmonary events.

## Methods

This cross-sectional study was deemed exempt from review by the Wayne State University Institutional Review Board because it did not meet institutional criteria for human participant research owing to the use of publicly available deidentified data. The study followed the Strengthening the Reporting of Observational Studies in Epidemiology (STROBE) reporting guideline.

For EPA-designated sensitive individuals, we estimated the number needed to treat (NNT) to prevent 1 serious health consequence, defined as an atherosclerotic cardiovascular disease (ASCVD) event (fatal or nonfatal myocardial infarction [MI] or stroke) in patients with preexisting ASCVD (and in the general population) and 1 pulmonary exacerbation in patients with asthma or chronic obstructive pulmonary disease (COPD). Treatment was defined as following the AQI activity guidance for PM_2.5_.^2^ Secondarily, we estimated the NNT values in proposed new categories of patients at even higher risk following more aggressive activity restrictions. These individuals were defined as patients who had experienced MI or heart failure (HF) within the prior 90 days and patients with higher-risk asthma or COPD experiencing 2 exacerbations per year.

### Modeling Assumptions and Calculations

Since the analysis was cross-sectional for any given single day, rather than a prospective risk assessment with follow-up over time, a cross-sectional modeling approach assessing the absolute frequency of events in a population per single day was used. This method should provide reasonable estimations at a population level. First, the baseline absolute event rate per day in a population of 1 million people was estimated. For patients with ASCVD, the most commonly estimated event rate is 2% per year.^11^ Therefore, we used the following formula: (0.02/365) × 1 000 000 = 54.79 events per day per million people. Because it is unlikely for adults aged 45 to 75 years in the general adult population without a prior diagnosis of asthma or COPD to have a serious pulmonary disease exacerbation, we only estimated the risk of ASCVD events because these do occur in seemingly healthy adults. Although rates vary substantially across calculated 10-year ASCVD risk scores (eg, 1%-20%), the mean rate across the entire population of adults aged 45 to 75 years without existing heart disease is 0.3% per year.^12^ Roughly half of patients with asthma or COPD have 1 exacerbation per year; thus, we used an absolute annual rate of 0.5 events.^13,14,15,16^

Second, excess absolute ASCVD and pulmonary event rates per day per million people were calculated by multiplying baseline daily event rates by the increases in risks due to elevations in exposures to PM_2.5_ at each AQI level. We used a monotonic 1% increase in risk per day for every 10-μg/m^3^ elevation above a good AQI (corresponding to a level of 12 μg/m^3^). The acute exposure-response (cardiopulmonary events or hospitalizations and mortality) relationship varies modestly across populations, study years, locations, pollution sources, and underlying cohort susceptibility (eg, age, race and ethnicity, socioeconomic status, and comorbidities); however, the risk we selected is well within the range of reported results.^17,18,19,20,21,22,23^ The counterfactual concentration of 12 μg/m^3^ was selected as the most logical comparison because it represents the desired annual air quality standard. The excess ASCVD and pulmonary event rates per day due to PM_2.5_ exposure were calculated for each AQI at the lower threshold cutoff level. As an example, the PM_2.5_ level is 36 μg/m^3^ for an AQI of 101. We subtracted 12 from 36 to yield an increase in exposure of 24 μg/m^3^, which corresponds to an approximately 2.4% increase in ASCVD or pulmonary events per day at this exposure level. For patients with ASCVD at this exposure, the calculation is as follows: 54.79 × 0.024 = 1.32 excess events per day per million people.

Third, we made plausible estimates of the 24-hour exposure reduction incurred by following the activity guidance. The estimates were made as recommended for the specific patient populations at each AQI level (defined in Tables 1 and 2).^2^

**Table 1.** NNT to Prevent Adverse Health Events From Ambient PM_2.5_ by Following AQI Guidance

Recommendation by group^a^	Exposure reduction, %			NNT			PNT, %
	Low estimate	Mean	High estimate	Low estimate	Mean	High estimate	
**Unhealthy for sensitive groups (AQI, 101-150; PM_2.5_, 35.5-55.4 μg/m^3^)**								
General public							
None	NA	NA	NA	NA	NA	NA	NA
Adults with ASCVD							
Decrease prolonged or heavy exertion outdoors	10	15	20	7 604 167	5 069 444	3 802 083	20.3
Adults with asthma or COPD							
Decrease prolonged or heavy exertion outdoors	10	15	20	304 167	202 778	152 083	5.3
**Unhealthy (AQI, 151-200; PM_2.5_, 55.5-150.4 μg/m^3^)**								
General public							
Decrease prolonged or heavy exertion outdoors	10	15	20	27 651 515	18 434 343	13 825 758	7.1
Adults with ASCVD							
Avoid prolonged or heavy exertion outdoors	20	25	30	2 073 864	1 659 091	1 382 576	6.6
Adults with asthma or COPD							
Avoid prolonged or heavy exertion outdoors	20	25	30	82 955	66 365	55 303	0.2
**Very unhealthy (AQI, 201-300; PM_2.5_, 150.5-250.4 μg/m^3^)**								
General public							
Avoid prolonged or heavy exertion outdoors	20	25	30	4 376 499	3 501 199	2 917 666	1.4
Adults with ASCVD							
Avoid all physical activities outdoors	30	35	40	437 650	375 128	328 237	1.5
Adults with asthma or COPD							
Avoid all physical activities outdoors	30	35	40	17 506	15 005	13 129	0.04
**Hazardous (AQI, 301-500; PM_2.5_, 250.5-500.4 μg/m^3^)**								
General public							
Avoid all physical activities outdoors	30	35	40	1 696 885	1 454 473	1 272 664	0.6
Adults with ASCVD							
Remain indoors or keep activity levels low	45	50	55	169 869	152 720	138 836	0.6
Remain indoors or no activity and use a PAC	70	75	80	109 085	101 813	95 450	0.4
Adults with asthma or COPD							
Remain indoors or keep activity levels low	45	50	55	6788	6109	5553	0.02
Remain indoors or no activity and use a PAC	70	75	80	4363	4073	3818	0.01

Abbreviations: AQI, air quality index; ASCVD, atherosclerotic cardiovascular disease; COPD, chronic obstructive pulmonary disease; NA, not applicable; NNT, number needed to treat; PAC, portable air cleaner; PM_2.5_, fine particulate matter less than 2.5 μm in diameter; PNT, population needed to treat.

^a^ Calculations for PM_2.5_ are as follows: unhealthy for sensitive groups, 36 μg/m^3^; unhealthy, 56 μg/m^3^; very unhealthy, 151 μg/m^3^; and hazardous, 251 μg/m^3^.

**Table 2.** NNT to Prevent Events From Ambient PM_2.5_ by Following AQI Guidance in Proposed Very High-Risk Patients

Recommendation by group^a^	Exposure reduction, %			NNT			PNT, %
	Low estimate	Mean	High estimate	Low estimate	Mean	High estimate	
**Unhealthy for sensitive groups (AQI, 101-150; PM_2.5_, 35.5-55.4 μg/m^3^)**							
Adults who experienced MI or HF within the prior 90 d							
Avoid all physical activities outdoors	30	35	40	50 000	42 857	37 500	2.1
Remain indoors or keep activity levels low	45	50	55	33 333	30 000	27 273	1.5
Adults with high pulmonary event risk^b^							
Avoid all physical activities outdoors	30	35	40	25 347	21 726	19 010	0.06
Remain indoors or keep activity levels low	45	50	55	16 898	15 208	13 826	0.04
**Unhealthy (AQI, 151-200; PM_2.5_, 55.5-150.4 μg/m^3^)**							
Adults who experienced MI or HF within the prior 90 d							
Avoid all physical activities outdoors	30	35	40	27 273	23 377	20 455	1.17
Remain indoors or keep activity levels low	45	50	55	18 182	16 364	14 876	0.82
Adults with high pulmonary event risk							
Avoid all physical activities outdoors	30	35	40	13 826	11 851	10 369	0.03
Remain indoors or keep activity levels low	45	50	55	9217	8295	7541	0.02
**Very unhealthy (AQI, 201-300; PM_2.5_, 150.5-250.4 μg/m^3^)**							
Adults who experienced MI or HF within the prior 90 d							
Remain indoors or no activity and use a PAC	70	75	80	3700	3453	3237	0.17
Adults with high pulmonary event risk							
Remain indoors or no activity and use a PAC	70	75	80	1876	1751	1641	0.005
**Hazardous (AQI, 301-500; PM_2.5_, 250.5-500.4 μg/m^3^)**							
Adults who experienced MI or HF within the prior 90 d							
Remain indoors or no activity and use a PAC	70	75	80	2152	2008	1883	0.1
Adults with high pulmonary event risk							
Remain indoors or no activity and use a PAC	70	75	80	1091	1018	954	0.003

Abbreviations: AQI, air quality index; HF, heart failure; MI, myocardial infarction; NNT, number needed to treat; PAC, portable air cleaner; PM_2.5_, fine particulate matter less than 2.5 μm in diameter; PNT, population needed to treat.

^a^ Calculations for PM_2.5_ are as follows: unhealthy for sensitive groups, 36 μg/m^3^; unhealthy, 56 μg/m^3^; very unhealthy, 151 μg/m^3^; and hazardous, 251 μg/m^3^.

^b^ Patients with 2 events per year.

Fourth, at each AQI, the absolute excess event rate per day per million people was multiplied by the estimated percentage in exposure reduction to provide the number of events prevented per day per million people. This presumes that the exposure reduction by the activity change follows the same exposure-response relationship of a 1% decrease in risk per day for every 10-μg/m^3^ lowering of PM_2.5_. For patients with ASCVD at an AQI of 101, the calculation is as follows: 1.32 × 0.15 (ie, a 15% reduction in exposure) = 0.198 events prevented per million people per day.

Fifth, the final NNT was calculated as the reciprocal of the number of events prevented per day per million people multiplied by 1 million. For patients with ASCVD at an AQI of 101, the calculation is as follows: (1/0.198) × 1 000 000 = 5 069 444 people.

Sixth, the process was repeated for proposed patient subgroups with a very high risk of pulmonary events. The total event rate (emergency department visits, rehospitalization, or mortality) within 90 days after MI or HF hospitalization was estimated at the high end of available data at 25% to provide optimistic NNT values.^24,25^ Patients with a very high risk of pulmonary events were defined as having 2 exacerbations per year.

Finally, the percentage of the (relevant) population needed to treat to prevent 1 event was calculated by dividing the NNT by the estimated number of people in each category using available data as follows: general adult public (260 million), adults with asthma (26 million) or COPD (12.5 million), all cardiovascular diseases (25 million), and annual number of patients post MI (800 000) or HF (1.2 million) admission.^26,27,28,29^

### Statistical Analysis

This study used Excel, version 2311 (Microsoft Corp). Analysis was conducted between August 1, 2023, and January 31, 2024.

## Results

The NNT to prevent ASCVD events was high for the general population and for patients with ASCVD across all AQI strata. The range of values was comparatively lower to prevent pulmonary events among adults with lung disease (Table 1). During the most common scenarios when AQIs ranged from 101 to 200,^9^ the NNT to prevent a serious disease event for the general population (>18 million), patients with ASCVD (approximately 1.6-5 million), and adults with lung disease (approximately 66 000-202 000) remained very high. In our proposed new categories involving even higher-risk patients taking more aggressive actions, each NNT was markedly lower, albeit the pool of individuals was also smaller (Table 2).

## Discussion

Reducing air pollution prevents cardiopulmonary events and saves lives.^5,6,7,8^ Fortunately, air quality has improved across the US over prior decades.^5,6,7,8,9,10^ An AQI greater than 100 for PM_2.5_ is now relatively uncommon. Data from the EPA show that from 2008 to 2022, an AQI greater than 100 only occurred from 67 to 279 days per year cumulatively among 35 major cities.^9^ During 2022, an AQI greater than 100 was reported on 1145 days in total across 3143 counties (ie, less than once per day across the entire US).^30^ Most occurrences were repeat events confined to more highly pollutant locations (eg, southern California), whereas many counties were not affected. The majority (96%) were also limited to the unhealthy for sensitive individuals (67.5%) and unhealthy (28.4%) categories.^30^ Taken in this context, our results question the relevance, practicality, and potential effectiveness of current AQI recommendations. Although an acceptable NNT is debatable for low-risk strategies following precautionary principles, current AQI recommendations would call for more than 5 million people with ASCVD (approximately 20% of all patients living in the entire US) to adhere to activity restrictions to prevent a single ASCVD event on pollution days occurring, on average, once (or a few times) per year in major cities. Even when AQI levels rarely reach unhealthy levels, more than 1.6 million patients with ASCVD and more than 18 million adults in the general population would need to follow the guidance. Although the NTT results in this study were lower for patients with asthma or COPD, they remained above 200 000 during the majority of action days (AQI, 101-150). During rare occasions, PM_2.5_ levels can reach hazardous designations. Only under these extreme scenarios (most of which are seasonal and due to wildfires)^31^ does the NNT fall below 10 000 and 150 000 for patients with lung disease and ASCVD, respectively. Even without considering imperfect public awareness or adherence to activity guidance,^32^ the population health benefits of the current recommendations are questionable. Put into context, many of the NNT values, including all of the results for the general public, exceed the median population (821 725) of the largest 143 counties in the US.^33^

We focused solely on AQI activity guidance for PM_2.5_ rather than for ozone or total AQI (highest of either PM_2.5_ or ozone) for several reasons. Most important, PM_2.5_ has a 10-fold greater total adverse public health effect than ozone.^3,4^ Second, PM_2.5_ poses risks of both cardiovascular and pulmonary diseases, whereas the effects of ozone on heart health are less robust. Our approach allowed us to assess whether AQI activity guidelines were associated with a reduction in both cardiovascular and pulmonary events. Elevations in ozone are also highly seasonal and, thus, AQI increases are limited to summer months throughout much of the US. Finally, the implications of the findings are more evident and messaging is clearer when focusing on a single pollutant rather than the total AQI. Future studies can reproduce our approach for ozone or total AQI.

We also focused on 2 important sensitive groups of patients as designated by the EPA: patients with cardiovascular disease and patients with pulmonary disease.^2^ Cardiopulmonary events are leading causes of death and account for more morbidity and mortality than all other diseases.^29,34^ The rates of ASCVD and pulmonary events are also well described among the germane populations. This allowed for reasonable estimations of the NNT as performed herein. Estimations in the general population were mostly provided for illustrative purposes and suggest the enormous NNT for otherwise healthy people. Future studies can replicate our approach in other sensitive groups.

Our findings in no way discredit the vital public health importance of AQIs and air pollution regulations per se. In fact, the unclear benefits of individual-level actions serve to reemphasize the utmost importance of continuing to improve national ambient air quality. Nonetheless, we believe the current approach of the AQI activity guide warrants reconsideration or updating. Our intent was not to solve this complex issue here. However, we have provided one example of how targeting very high-risk populations could be an effective approach or supplemental recommendation. After admission for acute coronary syndrome and HF, patients have an extremely high rate of recurrent events within only a few months, which explains the markedly lower NNT. There are likely many further modifications that could be helpful (eg, tailoring interventions to the calculated ASCVD risk score and COPD stage or number of exacerbations per year).

### Strengths and Limitations

A strength of our study is that it is the first, to our knowledge, to evaluate the public health relevance of AQI activity guidance by estimating the NNT in various populations to prevent serious cardiopulmonary events. Our findings provide important evidence that the current approach may require revisions.

This study also has some limitations. The main limitation is that our modeling estimations were dependent on several assumptions. A sound evidence base exists for most of the factors selected; however, the reductions in exposures provided by activity recommendations are less certain. Their effectiveness is likely highly variable and affected by a number of factors such as geographic region, urbanicity, local pollution sources, season, time spent outdoors, outdoor activities, and commute times in traffic.^3^ Awareness and seriousness of adherence to the recommendations is also important.^32,35,36,37^ We therefore provided an estimated low-end and high-end range of plausible exposure reductions (and corresponding NNT values), which should help account for this intrinsic variability. It is important to note that the final NNT results were not highly sensitive to variations in exposure reductions and remained within similar magnitude ranges irrespective of estimations. This is because the high NNT results are primarily explained by the low baseline frequency of serious health events on any given day in the populations (eg, 8 ASCVD events per million people per day in the general population) combined with the small increase in absolute risks due to single-day exposures to PM_2.5_ (eg, 0.36 ASCVD excess events per million people per day in the general population at an AQI of 50-100).

The likely source of greatest controversy in our study approach is that we were unable to find any scientific studies that demonstrate clinical personal-level PM_2.5_ exposure reductions that occur in response to each of the recommended activity restrictions. Most of the activity changes consist of rather modest alterations and are unlikely to yield major exposure reductions over a 24-hour average period. As such, we believe the estimations we used represent optimistic degrees of exposure reductions. We chose to be liberal in this regard to show the most positive potential outcome of following AQIs. We recognize there is a degree of uncertainty with the exposure reductions we estimated; therefore, we provided results using a range of values (including both low- and high-end estimations) of plausible benefits incurred from the actions. The recommendations for hazardous AQI levels also suggest considering the use of a portable air cleaner. The exposure reductions provided by staying indoors and using a portable air cleaner are better described at around 75% as we estimated.^3^ Most of these rare events are due to wildfires and not human-made air pollution.^31^

Although we indirectly accounted for variable adherence to activity recommendations in our methods by providing a plausible range of exposure reductions, our study did not consider imperfect public awareness. There is some literature that suggests both of these issues are affected by underlying comorbidities (eg, asthma) and prevailing poor air quality.^32,35,36,37^ Although awareness would influence the ultimate public health benefits, it does not directly affect the NNT calculation. Several household-related factors (eg, indoor penetration of ambient PM_2.5_, air conditioning and filtering, and indoor sources of exposures) also influence effectiveness in terms of how activity changes translate into exposure reductions.^38^ People spend most of their time (eg, 90%) indoors and the penetrance of outdoor ambient PM_2.5_ often exceeds 50%. In addition, various indoor sources (eg, cooking) can make indoor concentrations higher than ambient levels, as we have found in our studies in urban Detroit.^39^ Together, these considerations suggest that the range of exposure reductions we estimated could be overly optimistic. As with all methods applied to assess interventions, there are limitations to using the NNT.^40,41,42^ The main concern with our study is that the treatment duration is intrinsically limited to a single day. In most trials, the NNT is calculated per year or over the full duration of treatment and, as such, the values are markedly lower (eg, dozens to hundreds of people). Given the low absolute risk for adverse events on any given day in a population, the NNT is bound to be much higher in our analysis and not comparable to medical interventions (eg, cholesterol or blood pressure lowering).^40,42^ Nevertheless, AQI activity recommendations are specifically provided on a per-day basis; therefore, we believe our approach is well justified and borrows directly from public health guidance.^2^ Studies using other methods may provide complementary findings that, taken together with our results, could allow for a more holistic picture of the public health relevance.^42^

It is further possible that less severe health effects (eg, angina or dyspnea) may also be reduced by activity changes and that we failed to account for these benefits when calculating the NNT. However, this remains unproven, the symptoms are subjective and more difficult to accurately quantify, and the public health benefits are of less clear significance. It is also difficult to directly compare the lower NNT values in patients with lung disease compared with ASCVD. Pulmonary events (eg, COPD exacerbations) occur 10 to 15 times more frequently per year than cardiovascular events. Although pulmonary events are serious, they do not represent as major of an overall threat to health as a typical MI or stroke. We also could not consider the potential for theoretical harms from the daily promulgation of AQIs, such as inappropriate anxiety or concern vs disengagement due to “information overload.” Future studies assessing the risk-to-benefit ratios and economic costs (eg, missed work or school) would be helpful.

A last issue to consider is the heterogeneity of health risks posed by PM_2.5_, such as those due to health disparities. Adults living in lower socioeconomic communities are more highly exposed (vulnerable) and might also be more susceptible to adverse health effects.^43,44,45^ Our NNT results represent the averages for the entire populations evaluated. It is implicit that certain subgroups within every population will be at lower or higher risks following exposures than the mean; however, the calculated NNT values will only modestly vary (for the same reasons as previously described). Finally, PM_2.5_ exposure also poses risk to children’s respiratory health, largely from respiratory conditions (eg, asthma, infections).^46^ Although we only modeled NNT values in adults, the usual frequency of pulmonary events in children (eg, average of 0.5 asthma exacerbations per year) and exposure-response associations are similar to adults with lung disease. The NNT values will therefore be approximately the same.

## Conclusions

The results of this cross-sectional study suggest that the number of people who must successfully follow AQI activity recommendations per day to prevent serious cardiopulmonary consequences from PM_2.5_ exposure under most daily scenarios is very high. Our findings question the public health relevance of the current approach, and they also highlight the need for robust debate exploring new strategies to enhance the effectiveness of AQI guidance given its immense societal and public policy importance.

## Notes

Supplement.
Data Sharing Statement
