# Supplementary material for: Public Health Relevance of US EPA Air Quality Index Activity Recommendations
Source: JAMA Netw Open. 2024 Apr 8;7(4):e245292. doi: 10.1001/jamanetworkopen.2024.5292 (PMC11002695; doi:10.1001/jamanetworkopen.2024.5292)
Supplement: Supplement. — Data Sharing Statement [file jamanetwopen-e245292-s001.pdf]

Data Sharing Statement

Brook. Public Health Relevance of US EPA Air Quality Index Activity Recommendations. *JAMA Netw Open*. Published April 08, 2024. doi:10.1001/jamanetworkopen.2024.5292

Data
Data available: Yes
Data types: Data (not involving human participants)
How to access data: upon request - brook@wayne.edu
When available: With publication

Supporting Documents
Document types: Statistical/analytic code
How to access documents: upon request - brook@wayne.edu
When available: With publication

Additional Information
Who can access the data: anyone upon reasonable request
Types of analyses: any purpose
Mechanisms of data availability: without investigator support
Any additional restrictions: none
